# Effects of Cold-Surge-Induced Nearshore Seawater Icing on the Eukaryotic Microalgal Community in Aoshan Bay, Qingdao

**DOI:** 10.3390/microorganisms11010108

**Published:** 2022-12-31

**Authors:** Haizhen Bian, Xin Guo, Yanqiang Xu, Yubin Hu

**Affiliations:** Institute of Marine Science and Technology, Shandong University, Qingdao 266237, China

**Keywords:** sea ice, cold surge, low temperature, eukaryotic microalgae, microalgal community, mid-latitude, Aoshan Bay

## Abstract

Climate change has led to frequent cold surges in mid-latitudes, resulting in sudden temperature drops and icing of nearshore seawater, which may be affecting the eukaryotic microalgal community. In this paper, we investigated the differences between a eukaryotic microalgal community in sea ice and in seawater during the seawater freezing, due to the cold surge in Aoshan Bay, Qingdao, China, in January 2021. The results showed that the eukaryotic microalgal community in the sea ice and in the seawater was similar in composition at the phylum and genus levels, but that its relative abundances differed. In the seawater, the eukaryotic microalgal genera were dominated by *Chaetoceros*, while its relative abundance was significantly lower in the sea ice, probably because the cold-surge-induced seawater icing existed only for a short period of time, and *Chaetoceros* had not yet adapted to the rapid environmental changes in the sea ice. The relative abundance of *Bathycoccus* in the sea ice was higher, and showed a significant positive correlation with nitrite and silicate, while the relative abundance of *Micromonas* in the sea ice was also significantly higher than in the seawater, which may be related to the elevated CO_2_ concentration in the sea ice. This study demonstrates that although the seawater icing due to the cold surge was short, it may have affected the seawater eukaryotic microalgal community, to a certain extent.

## 1. Introduction

Since the industrial revolution, human activities have emitted large amounts of carbon dioxide (CO_2_) into the atmosphere, causing a dramatic increase in atmospheric CO_2_ concentrations. This increase in atmospheric CO_2_ has in turn led to global temperature rise and climate change [1,2]. There has been a significant increase in the occurrence of cold surges in Eurasia over the past 20 years, which is closely related to the decrease in Arctic sea ice due to rising temperatures [3,4,5,6]. Cold surge outbreaks are accompanied by extreme weather events such as abnormally low temperatures, freezing rain, and blizzards [7,8]. In addition, a sudden drop in temperature also creates conditions for seawater icing in the mid-latitudes, resulting in microorganisms having to adapt to the rapid environmental changes and the shift of their metabolism [9,10]. During the process of seawater icing, some salts will be enriched in the sea ice, forming brines with high salt concentrations [11,12]. Thus, the high-salinity brine in sea ice provides a unique environment for the establishment of microorganisms, including bacteria and algae from seawater, and other communities [13,14]. The activity of microbial eukaryotic microalgae in sea ice is influenced by environmental variables, such as temperature, salinity, nutrient concentrations and partial pressure of carbon dioxide (*p*CO_2_), all of which differ significantly from the seawater situation [15,16]. Eukaryotic microalgae in high-latitude waters may have adapted to the low temperature and sea ice environment [17], and some of them exhibit high biological activity in sea ice [18]. In contrast, a eukaryotic microalgal community in mid-latitude nearshore waters may not have developed effective coping strategies for sea ice formation, especially when sea ice formation and melting occur within a short period of time. Therefore, it is important to investigate how eukaryotic microalgal communities in mid-latitude waters respond to sea ice formation caused by cold surges.

Eukaryotic microalgae, as microscopic organisms prevalent in the global ocean, play an irreplaceable role in carbon cycling and in mitigating global warming [19,20,21]. In the context of global warming and climate change, it is unclear how a eukaryotic microalgal community in seawater changes during sudden cooling and icing caused by cold surges in mid-latitudes, where sea ice formation has rarely been observed in the past; therefore, it is important to study the effects of mid-latitude nearshore sea ice formation on a eukaryotic microalgal community. In this study, we investigated the differences in a eukaryotic microalgal community, between sea ice and seawater, in the coastal waters of Qingdao, China, after the cold surge outbreak in January 2021, in order to understand the effects of seawater icing on eukaryotic microalgal communities in mid-latitude coastal waters.

## 2. Materials and Methods

### 2.1. Station and Sample Collection

**Figure 1:**
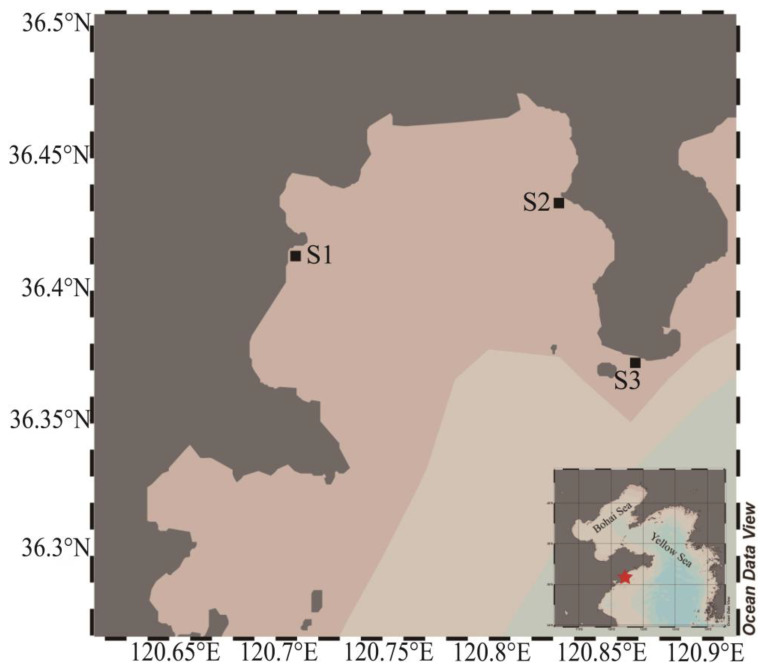
Map of the sampling location in Aoshan Bay, Qingdao. Black square indicates the sampling stations. Figure was generated using Ocean Date View (v 5.5.2) [24].

The study was performed in Aoshan Bay, a semi-enclosed bay in Qingdao, China. Due to its geographical location and climatic conditions, the minimum temperature in Aoshan Bay in winter is usually above −5 °C [22], and seawater seldom freezes; however, in January 2021, a cold surge lasting for 3 days in East Asia caused the minimum temperature in the bay to reach −14 °C, which resulted in extensive ice formation in the nearshore seawater of the bay, and the sea ice lasting for 7 days before melting. During this period, sea ice and seawater samples were collected, to investigate changes in the eukaryotic microalgal community. Samples were taken from three locations, two of which (S1 and S2) were located in the inner bay, while one was in the mouth of the bay (S3) (Figure 1). The sea ice thickness was around 30~50 mm, and the sea ice temperature was measured in situ by inserting a needle temperature probe (TP101, Tejiate, Shenzhen, China), with an accuracy of ±0.1 °C. In addition, the sea ice samples were obtained by a stainless steel saw, and were collected in polypropylene bags, from which air was extracted using a vacuum pump (H1, Reelanx, Shenzhen, China) [23]. The seawater samples beneath the ice were collected using 1L-HDPE bottles.

### 2.2. Sample Processing and Analysis

The sea ice samples were brought to the laboratory, melted in the dark at room temperature, and processed immediately after complete melting. The salinity of the melted sea ice and seawater was measured with a salinometer (Orion star A212, Thermo Scientific, Waltham, MA, USA). The 150 mL of melted sea ice and seawater were filtered through 0.45-μm-pore-size membrane filters (Millipore, Burlington, MA, USA), and dispensed in 40-mL-high borosilicate brown glass bottles and 125 mL high-density polyethylene (HDPE) bottles (Nalgene, Waltham, MA, USA) for pH, dissolved inorganic carbon (DIC) and total alkalinity (TA) analyses, respectively, where pH and DIC samples were adequately overflowed, and a 0.2‰ saturated mercury chloride (HgCl_2_) solution was added, to inhibit biological activities.

The pH was measured by a bench-top pH meter (Star A211, Thermo Fisher Scientific, Waltham, MA, USA) in a constant temperature water bath at 25 ± 0.05 °C. The Orion glass electrode (8157BNUMD, Thermo Fisher Scientific, Waltham, MA, USA) was calibrated with pH NBS buffers (pH = 4.010, 6.865, 9.183 @ 25 °C Mettler Toledo, Zurich, Switzerland), with a precision of better than ±0.01. The measured pH was converted to the in situ condition, according to the temperature. TA was titrated with hydrochloric acid via an automatic potentiometric titrator (T960E, Hanon, Jinan, China), with the concentration of hydrochloric acid being calibrated against certified reference material (CRM, Batch 178, Dickson lab, South Gate, CA, USA); the water sample was acidified to a pH below 3, and titration data in the pH range of 3 to 3.5 were obtained and processed by means of the Gran method. The measurement precision in TA was within ±2 µmol·kg^−1^. DIC was measured by a non-dispersive infrared detector (AS-C5, Apollo SciTech, Newark, NJ, USA). Using high-purity nitrogen (99.999%) as a carrier gas, the CO_2_ generated by acidification of a 1 mL seawater sample was blown into the drying system (magnesium perchlorate powder). Certified reference material (CRM, Batch 178, Dickson lab, South Gate, CA, USA) was used for quality control, with a precision of ±2 µmol·kg^−1^. *p*CO_2_ was calculated using temperature, salinity, TA and DIC data by CO2SYS (v 2.1) software [25]. For calculation, the equilibrium constants of the carbonate system were from Mehrbach et al. (1973) [26], revised by Dickson and Millero (1987) [27]; The dissociation constant of bisulfate (HSO_4_^−^) and [B]_T_ were adopted from Dickson (1990) [28] and Uppström (1974) [29], respectively. 

Next, 30 mL of melted sea ice and 30 mL of seawater were each filtered with a 0.45-μm-pore-size membrane filter (Millipore, Burlington, MA, USA), and the filtrate was stored at −20 °C for nutrient analysis. Nutrient concentrations—including nitrite, ammonium, silicate and phosphate—were measured, using a continuous-flow analyzer (AA3, SEAL analysis Ltd., Norderstedt, Germany), according to the classical colorimetric method [30]. The detection limit for all channels was 0.1 µmol·kg^−1^. DNA samples were collected by filtering melted sea ice through a 0.2-µm-pore-size polycarbonate membrane (Millipore, USA), and doing likewise for seawater, after which, the polycarbonate membranes were stored in cryotubes at −80 °C until DNA extraction. 

Brine salinity (S_brine_) was calculated from the ice temperature, by the formula: S_brine_ = 1000/[1 − (54.11/T)] [31]. The sea ice pH, TA, DIC and nutrient data were normalized to brine concentration, to correct for dilution during melting, and normalized salinity (C_brine_) was calculated according to the equation (C_brine_) = C_bulk_ (S_brine_/S_bulk_), where C_bulk_ was the measured concentration of bulk sea ice, S_brine_ was the brine salinity, and S_bulk_ was the measured salinity of the melted ice [31].

### 2.3. DNA Extraction, Amplification and Sequencing

DNA was extracted from 0.2-µm-pore-size polycarbonate filter membranes (Millipore) using DNeasy PowerSoil Kit (Qiagen, Hilden, Germany) [32]. Eukaryotic microalgae 18S rRNA sequences were amplified using primers 528F (5’-GCGGTAATTCCAGCTCCAA-3’) and 706R (5’-AATCCRAGAATTTCACCTCT-3’). The V4 region of the eukaryotic microalgae sequences was sequenced, using an Illumina Hiseq 2500 platform (Novogene, Tianjin, China).

### 2.4. Bioinformatics and Statistical Analysis

The raw sequences were demultiplexed and quality-filtered by the bioinformatics analysis software QIIME2 [33], where amplicon sequencing variants (ASVs) were generated using DADA2, and were classified according to the SILVA 138 database [34]. In order to better analyze the diversity and community structure of eukaryotic microalgae, this study removed non-algal ASVs, including Ascomycota, Arthropoda, Centrohelida, Mucoromycota and Vertebrata, as well as unclassified data. The number of sequences per sample ranged from 21,217–39,822, and were uniformly normalized to 21,217 sequences, to avoid potential bias due to sequencing depth. A total of 348 ASVs were obtained for downstream analysis. The α-diversity, including Shannon–Weiner (Diversity) and the Pielou (Evenness) index [35], was calculated for each sample, using the vegan R package [36]. Differences between the eukaryotic microalgal community in seawater and sea ice were analyzed by *t*-test, prior to which the data were tested for normality and homogeneity of variance in GraphPad Prism (v 8.4.3). The compositions of the eukaryotic microalgal community in the seawater and in the sea ice at the genus level were calculated, and presented using the vegan and pheatmap packages in R [37]. Differences in the eukaryotic microalgal community in the sea ice and in the seawater were explored, using principal coordinate analysis (PCoA analysis) based on Bray–Curtis distances. The variability of the eukaryotic microalgal community in the sea ice and in the seawater at the genus level was examined using STAMP [38]. The 12 most abundant genera were analyzed for correlation with environmental factors using Spearman correlation, and significant differences between the dominant genera of the eukaryotic microalgal community and environmental factors were demonstrated, using the ggplot2, pheatmap and Hmisc packages in R [37,39,40].

## 3. Results

### 3.1. Environment Parameters

As shown in Table 1, the salinity of the sea ice brine varied from 35.6 to 55.8, with a mean of 43.5, and the salinity of the seawater varied from 32.2 to 40.4, with a mean of 35.2. The temperature in the seawater was about −1.1 °C, while the temperature in the sea ice varied from −3.2 to −2.0 °C. The salinity and *p*CO_2_ of the seawater at S1 were higher than those in the corresponding sea ice, while the pH and nutrient data were lower than those in the sea ice. The reason that the salinity and *p*CO_2_ at S1 were higher than those at the other two stations was probably due to the shallow water depth at this station, and the expulsion of salt during the sea ice formation leading to an increase in the salinity of the seawater under the ice, which in turn led to an increase in *p*CO_2_. Salinity, pH, *p*CO_2_ and nutrient data at S2 and S3 were all lower than those in the corresponding sea ice.

### 3.2. Eukaryotic Microalgal Diversity and Community Composition

**Figure 2:**
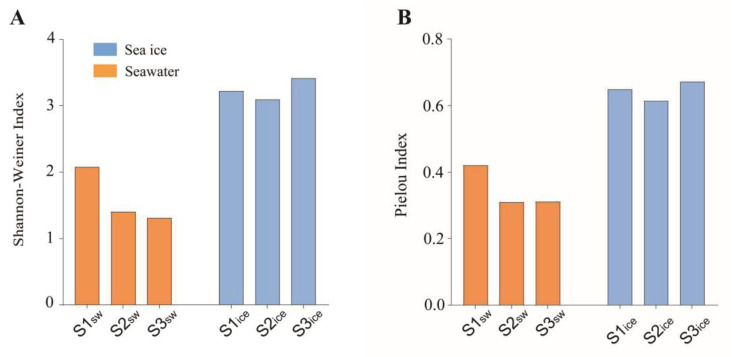

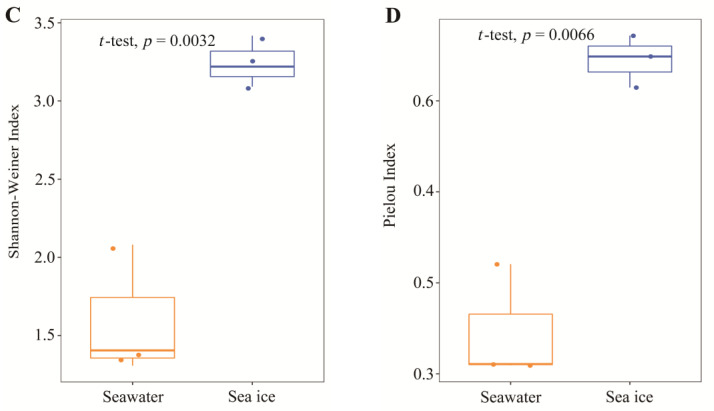
α-diversity of the eukaryotic microalgal community in the seawater and in the sea ice: Shannon’s index (**A**) and evenness (**B**); significance test of Shannon’s index (**C**) and evenness (**D**).

The Shannon–Weiner Index of the eukaryotic microalgal community was 2.08, 1.41 and 1.31 in the seawater, and 3.22, 3.09 and 3.42 in the sea ice at S1, S2 and S3, respectively (Figure 2A). The Pielou Index was 0.42, 0.31 and 0.31 in the seawater, and 0.65, 0.61 and 0.67 at S1, S2 and S3 in the sea ice, respectively (Figure 2B). The diversity and evenness of the eukaryotic microalgal community in the sea ice were significantly higher than in the seawater (Figure 2C,D) (*p* < 0.05).

**Figure 3:**
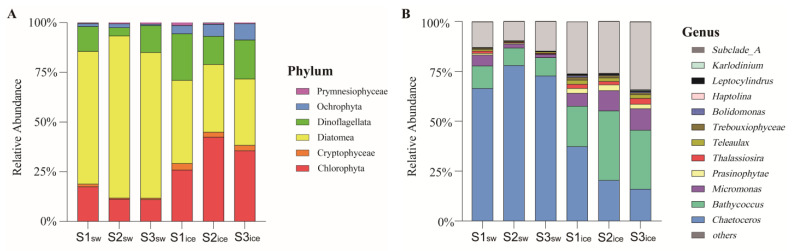
Relative abundance of the eukaryotic microalgal community in the seawater and in the sea ice at the phylum level (**A**) and genus level (**B**).

A total of six eukaryotic microalgal clades were identified, and the eukaryotic microalgal community in the sea ice and in the seawater was similar in composition at the phylum level (Figure 3A); only Diatomea was more dominant in the seawater samples than in the sea ice samples (*p* < 0.05), with a relative abundance accounting for 66–82% of the entire eukaryotic microalgal community in the seawater samples, while it only accounted for 33–42% in the sea ice samples, although the relative abundance was still high. The relative abundances of Dinoflagellata and Chlorophyta were higher in the sea ice samples, accounting for 14–24% and 26–43%, respectively, while the relative abundances in the seawater samples were lower, accounting for 4–14% and 11–18%, respectively. In addition, Prymnesiophyceae, Ochrophyta, and Cryptophyceae were also detected in the seawater and sea ice samples, and were more dominant in the sea ice samples, but their relative abundances were lower, accounting for 0.4–1.4%, 4.1–8.2% and 2.4–3.3%, respectively. The relative abundances of Chlorophyta, Ochrophyta and Cryptophyceae in the sea ice samples were significantly higher than those in the seawater (*p* < 0.05), while no significant difference was seen for Dinoflagellata and Prymnesiophyceae (*p* > 0.05).

At the genus level (Figure 3B), the eukaryotic microalgal community in the sea ice and in the seawater was similar in composition, but the dominant algal genus in the seawater was *Chaetoceros*, and the relative abundance of *Chaetoceros* in the seawater samples was significantly higher than that in the sea ice samples (*p* < 0.05). The relative abundance of *Chaetoceros* was highest at S2, at 78%, and decreased sequentially at S1 and S3, at 72% and 67%, respectively. The relative abundance of *Chaetoceros* in the sea ice decreased compared to that in the seawater, but it was still the most abundant algal genus at S1, at 37%, and lower at S2 and S3, at 20% and 16%, respectively. The relative abundance of *Bathycoccus* was only lower than *Chaetoceros* in the sea ice at S1, at 20%, and was highest at S2 and S3, at 35% and 30%, respectively; in the seawater, its relative abundance was significantly lower (*p* < 0.05), at 11%, 9% and 9% at S1, S2 and S3, respectively. The relative abundance of *Micromonas* was significantly higher in the sea ice than in the seawater (*p* < 0.05), at 6%, 10% and 11% in the sea ice at S1, S2 and S3, respectively, and 5%, 2% and 2% in the seawater, respectively.

### 3.3. Differences of Eukaryotic Microalgal Community in Seawater and Sea Ice

**Figure 4:**
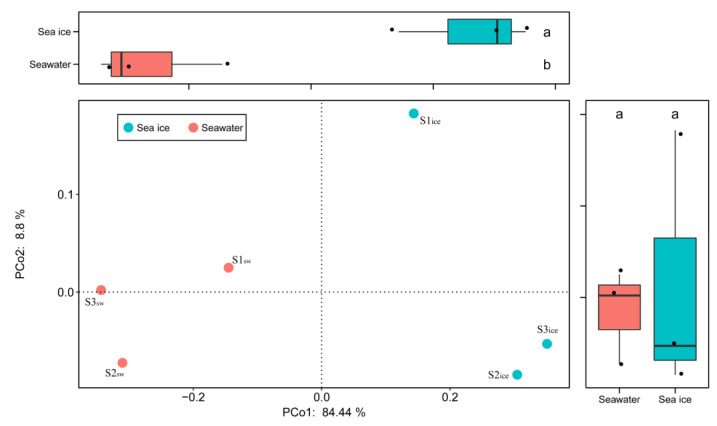
Principal coordinates analysis (PCoA) of Bray–Curtis distance for the eukaryotic microalgal community in the seawater and in the sea ice. Different letters (a and b) denote significant differences (*p* < 0.05).

Based on the Bray–Curtis distance analysis, PCoA was used to evaluate the overall differences between the sea ice and the seawater eukaryotic microalgal community (Figure 4). PCo1 and Pco2 explained 84.44% and 8.8%, respectively, of the differences among the six samples. The seawater samples were located in the negative half-axis of PCo1, while the sea ice samples were located in the positive half-axis of PCo1, indicating that the eukaryotic microalgal community was significantly clustered, due to the phase transition of the seawater icing.

**Figure 5:**
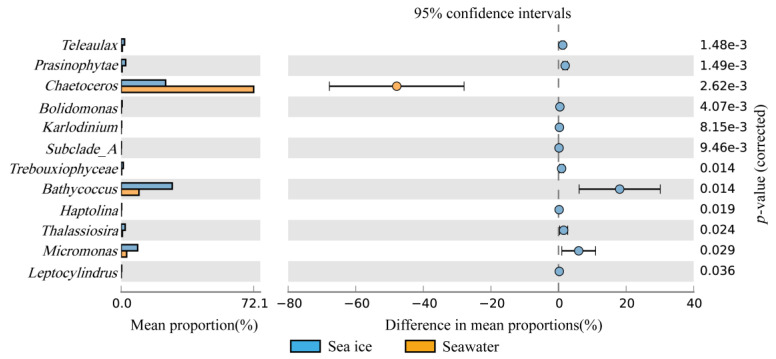
Comparison of genus-level differences between the seawater and the sea ice eukaryotic microalgal community (*t*-test in STAMP). Color: Eukaryotic microalgae in the seawater (orange) and in the sea ice (blue).

The results of STAMP analysis showed that at the genus level, *Chaetoceros* was relatively more abundant in the seawater, while *Prasinophytae*, *Teleaulax*, *Subclade_A*, *Trebouxiophyceae*, *Bolidomonas*, *Haptolina*, *Thalassiosira*, *Micromonas*, *Karlodinium*, *Leptocylindrus*, *Bathycoccus* were more abundant in the sea ice. The most significant differences in dominant genera between the seawater and the sea ice samples were *Chaetoceros*, *Bathycoccus* and *Micromonas* (*p* < 0.05) (Figure 5).

### 3.4. Correlation of Eukaryotic Microalgae Genera with Environmental Parameters

**Figure 6:**
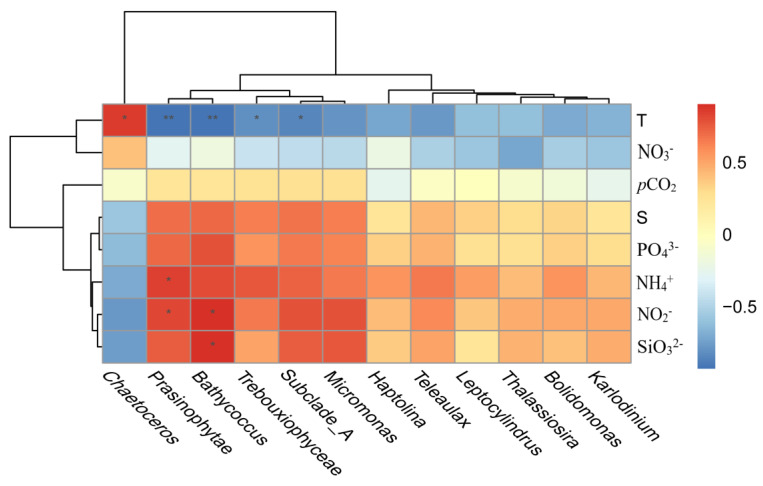
Spearman correlation matrix of eukaryotic microalgae algae genera and environmental parameters. Colors indicate correlation coefficients, with red indicating a positive correlation, and blue indicating a negative correlation. “*” represents significant correlation (*p* < 0.05) and “**” represents highly significant correlation (*p* < 0.01).

Among the 12 genera with the highest abundance screened for correlations with environmental parameters, the analysis revealed that *Chaetocerps* showed a significant positive correlation with temperature (*p* < 0.05); *Bathycoccus* showed a significant positive correlation with NO_2_^−^ and SiO_3_^2−^ (*p* < 0.05), and a highly significant negative correlation with temperature (*p* < 0.01); *Prasinophytae* was significantly positively correlated with NH_4_^+^ and NO_2_^−^ (*p* < 0.05), and highly significantly negatively correlated with temperature (*p* < 0.01); *Subclade_A* and *Trebouxiophyceae* were both negatively correlated with temperature (*p* < 0.05) (Figure 6).

## 4. Discussion

Cold surges cause nearshore seawater to freeze, and the phase change process of seawater icing can affect the metabolic activities of the eukaryotic microalgae, forcing them to adapt to a new lifestyle in sea ice. In this study, the similarity of eukaryotic microalgal community in the sea ice and in the seawater was higher than the effects arising from the spatial distance of the samples, and the difference in living environments due to the sea ice formation was an important factor in altering the eukaryotic microalgal community. In addition, this study found that the composition differences between the eukaryotic microalgal community in seawater and in sea ice at the phylum and genus levels were not significant, possibly due to the short existence of the sea ice, and because the microalgal cells in the sea ice may have still maintained their metabolic activities in the open water [10,42]. The differences in the eukaryotic microalgal community were mainly reflected in the relative abundance, e.g., in the seawater, the eukaryotic microalgal community had the greatest relative abundance of diatoms at the phylum level, while in the sea ice, the relative abundance of diatoms significantly decreased (*p* < 0.05). The relative abundance of *Chaetoceros* was greatest in the seawater at the genus level, but decreased significantly in the sea ice (*p* < 0.05).

*Chaetoceros* has been found to be a central algal genus in both seawater and sea ice in the Arctic, and the sea ice environment is also favorable for the growth of the sea ice diatom *Chaetoceros* [43,44]. In this study, *Chaetoceros* was also the most abundant algal genus in the surveyed area, but the relative abundance of *Chaetoceros* in the sea ice was significantly lower than in the seawater, which was different from the findings of the polar sea ice study. This difference may have been because our sampling site was located in mid-latitude, where the seawater does not normally freeze, and the diatoms may not have adapted to the dramatic environmental changes in a short period of time, due to the sudden cold surge that caused the freezing of the seawater. *Bathycoccus* was the dominant genus in the sea ice, and is also the main eukaryotic microalgae in polar regions [45,46]: it possesses two ecotypes, one of which adapts to environments with high nutrient concentrations in coastal waters, while the other adapts to environments with low nutrient concentrations in the open ocean [47,48]. Nutrient enrichment in sea ice brines due to seawater icing may stimulate the growth of *Bathycoccus* in sea ice, as evidenced by a positive correlation between *Bathycoccus* and nutrient salt concentrations, especially a significant correlation with nutrient NO_2_^−^ and SiO_3_^2−^ [49]. In addition to being the dominant genus, the relative abundance of *Micromonas* was significantly higher in the sea ice than in the seawater (*p* < 0.05), and it is also widely distributed in polar sea ice [50]. CO_2_ concentration has been shown to affect the development of the eukaryotic community [51]. In particular, the abundance of *Micromonas* will significantly increase at high CO_2_ concentrations [52]. As *p*CO_2_ in the sea ice was higher than in the seawater (except at S1), *Micromonas* exhibited a higher relative abundance in the sea ice at high CO_2_ concentrations.

## 5. Conclusions

Against the background of global climate change, nearshore seawater icing in mid-latitude waters is likely to become the new normal. In this study, we investigated the differences between a eukaryotic microalgal community in seawater and in sea ice in Aoshan Bay, Qingdao, after a cold surge. The compositions of the eukaryotic microalgal community in seawater at the phylum and genus levels were similar to those in the sea ice, but their relative abundances were significantly different, which may be related to the environmental differences caused by the phase change pressure of seawater icing. The eukaryotic microalgal community in the seawater was dominated by the diatom *Chaetoceros*; and the results of the reduced relative abundance of *Chaetoceros* in the sea ice differed from those in the Arctic study, probably because the sampling site of this study was in the mid-latitude coastal waters, where the existing time of seawater icing was short, and *Chaetoceros* may have not yet adapted to the rapid environmental changes. The eukaryotic microalgal community in the sea ice was dominated by *Bathycoccus* and *Micromonas*, which had more opportunities to grow in the sea ice with, respectively, higher nutrient and CO_2_ concentrations. Due to the limited investigation in this study, it is unknown how a eukaryotic microalgal community evolves during the formation and melting of sea ice induced by cold surges. Therefore, we suggest that future studies may consider conducting time series surveys, to understand the effects of cold surges on eukaryotic microalgal communities in mid-latitude waters more comprehensively.

## Figures and Tables

**Table 1:** Salinity (S), temperature (T), pH, *p*CO_2_ (μatm) and nutrient concentrations (µmol·kg^−1^) in the seawater and in the sea ice brine.

Sample	S	T (°C)	pH	*p*CO_2_	NO_2_^−^	NO_3_^−^	NH_4_^+^	SiO_3_^2−^	PO_4_^3−^
S1sw	40.4	−1.0	8.16	346	0.5	3.8	2.8	5.1	0.1
S2sw	33.1	−1.2	8.20	277	0.2	16.4	1.4	5.8	0.1
S3sw	32.2	−1.0	8.24	239	0.2	12.1	0.6	4.8	0.0
S1ice	39.1	−2.2	8.26	279	0.8	4.7	16.7	5.7	0.2
S2ice	55.8	−3.2	8.29	374	2.9	15.0	33.2	20.4	0.9
S3ice	35.6	−2.0	8.28	252	1.2	-	6.5	11.8	0.2

S1, S2 and S3 denote station names, and the subscript “sw” and “ice” denote data in the seawater and in the sea ice, respectively (some data are cited from Ren et al. [41]).

## Data Availability

Illumina sequences are available in the Sequence Read Archive (SRA) of the National Center for Biotechnology Information (NCBI), under BioProject accession number PRJNA908654.
